# Anti-PD-1 Treatment Restores Effector Function in Exhausted-like T Cells from Malignant Ascites of Ovarian Cancer Patients

**DOI:** 10.3390/cancers18162612

**Published:** 2026-08-13

**Authors:** Diana Luísa Almeida-Nunes, Ana Mendes-Frias, Mariana Nunes, Verónica Ferreira, Cláudia Lobo, Paula Monteiro, Miguel Henriques Abreu, Carla Bartosch, Claudia Nobrega, Ricardo Jorge Dinis-Oliveira, Ricardo Silvestre, Sara Ricardo

**Affiliations:** 1 Associate Laboratory i4HB, Institute for Health and Bioeconomy, University Institute of Health Sciences—CESPU, 4585-116 Gandra, Portugal; nunesdiana@msn.com (D.L.A.-N.); sara.ricardo@iucs.cespu.pt (S.R.); 2 UCIBIO—Applied Molecular Biosciences Unit, Toxicologic Pathology Research Laboratory, University Institute of Health Sciences (1H-TOXRUN, IUCS-CESPU), 4585-116 Gandra, Portugal; 3 Differentiation and Cancer Group, Institute for Research and Innovation in Health (i3S) of the University of Porto, 4200-135 Porto, Portugal; marianaoliveiranunes1992@gmail.com; 4 Life and Health Sciences Research Institute (ICVS), School of Medicine from University of Minho, 4710-057 Braga, Portugal; anamendesfrias@gmail.com (A.M.-F.); claudianobrega@med.uminho.pt (C.N.); 5 ICVS/3B’s—PT Government Associate Laboratory, 4710-057 Braga, Portugal; 6 School of Medicine and Biomedical Sciences (ICBAS), University of Porto, 4050-313 Porto, Portugal; 7 Department of Pathology, Portuguese Oncology Institute of Porto (IPO-Porto), 4200-072 Porto, Portugal; veronicaf@ipoporto.min-saude.pt (V.F.); claudia.lobo@ipoporto.min-saude.pt (C.L.); paula.monteiro@ipoporto.min-saude.pt (P.M.); carla.bartosch@ipoporto.min-saude.pt (C.B.); 8 Department of Medical Oncology, Portuguese Oncology Institute of Porto (IPO-Porto), 4200-072 Porto, Portugal; antonio.m.abreu@ipoporto.min-saude.pt; 9 Porto Comprehensive Cancer Center Raquel Seruca (PCCC), 4200-162 Porto, Portugal; 10 Cancer Biology & Epigenetics Group, Research Center of Portuguese Oncology Institute of Porto (CI-IPO-Porto)/Health Research Network (RISE@CI-IPO-Porto), Portuguese Oncology Institute of Porto (IPO-Porto), 4200-072 Porto, Portugal; 11 UCIBIO—Applied Molecular Biosciences Unit, Translational Toxicology Research Laboratory, University Institute of Health Sciences (1H-TOXRUN, IUCS-CESPU), 4585-116 Gandra, Portugal; 12 Department of Public Health and Forensic Sciences, and Medical Education, Faculty of Medicine, University of Porto (FMUP), 4200-319 Porto, Portugal; 13 FOREN—Forensic Science Experts, 1400-136 Lisboa, Portugal

**Keywords:** high-grade serous carcinoma, malignant ascitic fluid, tumor immune microenvironment, anti-PD-1, T cell exhaustion-like phenotype

## Abstract

**Background/Objectives**: High-grade serous carcinoma (HGSC) represents the most frequent subtype of epithelial ovarian cancer (OC) and is associated with malignant ascitic fluid (MAF), which fosters a pro-inflammatory microenvironment that supports tumor progression. Given its rich cellular and soluble content, MAF offers a valuable window into tumor–host interactions and disease dynamics. This study examines the immune landscape of MAF samples from 22 newly diagnosed treatment-naïve HGSC patients. **Methods**: Immune cell phenotypes and cytokine profiles were analyzed via flow cytometry. Patients were stratified according to time to death or recurrence (TDR), using a six-month interval from diagnosis to either documented recurrence or disease-specific death as the cutoff: TDR < 6 months defined as the worse prognosis group, whereas TDR ≥ 6 months defined the better prognosis group. The expression of immune checkpoint molecules on T cells and the effects of PD-1 blockade with Pembrolizumab were also assessed. **Results**: Patients with worst prognosis exhibited a marked pro-inflammatory cytokine milieu, with elevated levels of TNFα, IL-1β, IL-23, and IFNγ. Concurrently, their CD4^+^ and CD8^+^ T cells displayed evidence of a functional exhaustion-associated phenotype, marked by heightened expression of the inhibitory receptors TIM-3, PD-1, and LAG-3. Notably, treatment with Pembrolizumab (a PD-1 checkpoint inhibitor) significantly enhance T cell effector function. **Conclusions**: These results underscore a potential immunotherapeutic approach in anti-tumor immunity among selected HGSC patients, presenting a compelling direction for advancing OC treatment.

## Simple Summary

Ovarian cancer is often diagnosed at an advanced stage, when malignant ascitic fluid accumulates in the abdomen and creates an environment that helps cancer grow and spread. Understanding how the immune system behaves in this fluid may help identify patients with different clinical outcomes and reveal new treatment opportunities. In this study, we analyzed immune cells and inflammatory molecules in these malignant fluid samples from women with high-grade serous ovarian cancer. Patients with poorer outcomes showed higher levels of inflammation and signs of T cells exhaustion. Importantly, treatment with an immune checkpoint inhibitor was able to restore immune cell activity in laboratory tests. These findings improve our understanding of the relationship among inflammation, immune dysfunction, and disease progression and support the development of personalized immunotherapy strategies for selected patients with ovarian cancer.

## 1. Introduction

Ovarian cancer (OC) is among the most common and lethal gynecologic cancers, primarily due to a lack of specific symptoms and effective screening tools, which often leads to diagnosis at advanced stages [1]. This cancer is marked by rapid tumor growth and extensive spread throughout the peritoneal cavity [2]. The World Health Organization categorizes ovarian tumors into three main types: epithelial (accounting for nearly 90% of cases), germ cell (3%), and sex cord–stromal (2%) [3]. Representing approximately 70% of epithelial OC, high-grade serous carcinoma (HGSC) is the most frequent subtype and originates from secretory epithelial cells of the fallopian tube via a series of precursor lesions [4,5]. At diagnosis, HGSC often involves both ovaries and is associated with widespread peritoneal carcinomatosis, with the omentum being particularly affected [6,7]. While initially responsive to chemotherapy, a small subgroup (<10%) exhibits intrinsic resistance, rendering the disease platinum-refractory [8]. Despite initial clinical remission, approximately 75% of patients relapse within 3 years, and recurrent disease is generally incurable [8,9].

A hallmark of HGSC is the accumulation of malignant ascitic fluid (MAF) in the peritoneal cavity, which enables tumor cells to disseminate across the peritoneal and pelvic surfaces, enhancing metastatic potential [10]. MAF is pivotal in promoting tumor adhesion to the omentum and the serous membranes of peritoneal organs, thereby facilitating the formation of secondary lesions [11]. MAF comprises both a cellular component—tumor cells [12] and various non-tumor cells, such as fibroblasts, mesothelial cells, endothelial cells, adipocytes, and immune cells [13,14]—and an acellular component rich in tumor-promoting factors, including angiogenic and growth factors, bioactive lipids, cytokines, chemokines, and extracellular vesicles [15].

The tumor immune microenvironment (TIME) within MAF is shaped by the dynamic crosstalk between cancer cells and immune elements, which collectively reprogram innate and adaptive responses to favor tumor growth and metastasis. This communication occurs through direct cell–cell interfaces between cancer cells and stromal cells or indirect cytokine-mediated interactions and activating pathways that drive the aggressive and metastatic behavior of HGSC [8]. Among these factors, pro-inflammatory cytokines and chemokines exert paracrine and autocrine effects that modulate the tumor milieu [16].

Importantly, MAF promotes an immunosuppressive microenvironment, markedly different from the immune profiles observed in peripheral blood or solid tumors [17]. Within the leukocyte populations, anti-tumoral cells include CD8^+^ cytotoxic T cells and CD4^+^ T helper (Th) 1 cells, whereas pro-tumoral populations include myeloid-derived suppressor cells (MDSCs), tumor-associated macrophages (TAMs, especially presenting an M2-like phenotype), Th2 cells, and regulatory T cells (Tregs) [8]. These latter components contribute to tumor cell survival, progression, resistance to chemotherapy, and immune evasion [18,19].

Promoting robust anti-tumoral immune responses has emerged as a key strategy in HGSC [20]. Indeed, high levels of tumor infiltrating lymphocytes (TILs), specifically CD8^+^ T cells, correlate with improved patient outcomes [20]. However, the presence of TILs alone does not guarantee an effective immune response [21]. Continuous antigen exposure, suppressive immune cells, or chronic inflammation results in repeated T cell receptor (TCR) stimulation, gradually impairing T cell functions—an effect known as immune exhaustion [21,22]. Phenotypic changes in exhausted T cells include the increased expression of co-inhibitory receptors, such as T cell immunoglobulin and mucin-domain-containing protein 3 (TIM-3), programmed cell death protein 1 (PD-1), and lymphocyte activation gene 3 protein (LAG-3) [21]. Tumor or suppressive immune cells often express ligands for these receptors, dampening T cell activity and facilitating immune evasion [21]. Notably, immune exhaustion is reversible, offering opportunities for therapeutic intervention with immune checkpoint inhibitors, such as anti-PD-1 therapies (e.g., Pembrolizumab), which have shown potential across multiple cancer types [21].

This study provides a comprehensive characterization of MAF in HGSC patients at diagnosis by analyzing immune cell populations and the cytokine profile. Additionally, it investigates the extent to which this unique liquid microenvironment induces T cell exhaustion and explores the potential for functional restoration via PD-1 blockade. These findings reveal that patients with a poorer prognosis exhibit reduced percentages of T cells at diagnosis, with the majority of these cells exhibiting an exhausted phenotype. Notably, ex vivo stimulation with anti-PD-1 therapy restores enhanced T cell effector functions, increasing degranulation and TNFα and IFNγ production, highlighting its therapeutic potential and supporting a personalized approach to immunotherapy in HGSC.

## 2. Materials and Methods

### 2.1. Patient Recruitment

Twenty-two women diagnosed with HGSC were recruited following ethical rules according to Helsinki and by the Ethical Committee for Health of the Portuguese Institute of Oncology from Porto (IPO-Porto, Ref. 92R1/019) [23]. Written informed consent was obtained from all participants prior to their enrollment in the study. The MAF samples were collected at IPO-Porto and were part of the Biobank, which was established in 2019. This study was conducted between February 2021 and February 2024. Experienced pathologists performed the histopathological classification at the IPO-Porto Pathology Lab. The MAF total volume ranged from 0.5 to 4 L and was obtained at diagnosis before treatment initiation (from now on referred to as treatment-naïve). Patient status was defined based on the following: on death from OC/disease (DOD); alive and responsive to treatment but with disease (AWD); or alive without evidence of disease (AWoD). Time to death or recurrence (TDR) was defined as the number of months from diagnosis to either documented recurrence or disease-specific death, whichever occurred first. This metric reflects the period when patients remained free from recurrence or disease-related mortality. Of notice, treatment response refers to the post-therapy evaluation, while patient status and the TDR reflect longitudinal outcomes at the time of follow-up.

### 2.2. MAF Sample Collection and Separation into Cellular and Acellular Fractions

The patient’s samples were collected, and the full volume was centrifuged at 400 g for 10 min at 4 °C. Aliquots of the acellular fraction (supernatant) were stored at −80 °C for cytokine quantification. The resuspended pellet, the cellular fraction, was incubated with 5 mL of Red Blood Cell Lysis Solution (Miltenyi Biotec, Bergisch Gladbach, Germany, cat.no. 130-094-183; diluted 10-fold with deionized water) and incubated for 5 min with occasional shaking. The reaction was stopped by adding 30 mL of phosphate-buffered saline (PBS) and centrifuged at 400 g for 5 min. The MAF-derived cells were resuspended in freezing medium [90% fetal bovine serum (FBS; Biowest, Nuaillé, France, cat no. S181A) with 10% dimethyl sulfoxide (DMSO; AppliChem, Barcelona, Spain, cat no. A3672,0050)], aliquoted with ~2 million cells per vial, and stored at −80 °C.

### 2.3. Phenotypic Characterization of MAF-Derived Cells

MAF-derived cells were thawed and equally divided into four different antibody panels (Supplementary Table S1) to evaluate the following: (i) innate immune cells; (ii) adaptive immune cells; (iii) T cell exhaustion markers, and (iv) intracellular molecules. Cells were first incubated with the Zombie Green Fixable Viability kit (cat no. 423111, Biolegend, San Diego, CA, USA) for 20 min at 4 °C in the dark. For the surface stain, MAF-derived cells were incubated with the antibody mix for 20 min at 4 °C in the dark. For the intracellular stain that was required, the Foxp3/Transcription Factor Staining Buffer Set (Invitrogen, cat no. LTI 00-5523-00) was used according to the manufacturer’s instructions; in this case, the intracellular antibody mix was incubated for 20 min at room temperature in the dark. Samples were acquired on the LSRII flow cytometer (BD Biosciences, Milpitas, CA, USA) using the FACS DIVA Software, and data were analyzed using FlowJo software (V10, Tree Star, Woodburn, OR, USA). The gating strategies to characterize the immune cell populations are presented in Supplementary Figures S1–S3.

### 2.4. Treatment of MAF-Derived Cells with Anti-PD-1

MAF-derived cells were thawed in complete RPMI medium (RPMI-1640 medium supplemented with 2 mM glutamine, 10% FBS, 10 U/mL penicillin/streptomycin, and 10 mM HEPES; all from Gibco, Thermo Fisher Scientific, Grand Island, NY, USA) and centrifuged at 250 g for 5 min to remove DMSO. After resuspension in complete RPMI medium, the cells were allowed to recover overnight in the presence of 10 ng/mL of IL-2 (Peprotech, London, UK, cat no. 200-02) and 20 U/mL of Deoxyribonuclease I (DNAse-I; Sigma-Aldrich, St Louis, MO, USA, cat no. DN-25). After resting, the cells were washed and seeded at 2.5 × 10^5^ cells/mL (per well) in a 96-well U-shaped plate (Corning, NY, USA), with 10 ng/mL of IL-2 and 1000-fold diluted Cell Stimulation Cocktail (plus protein transport inhibitors; eBioscience, San Diego, CA, USA, cat no. 00-4975-93); to maximize the CD107 Pe-Cy7 (BioLegend, cat no. 328618) stain [24], 1.25 µL/200 µL of antibody was added to cells during the incubation period. The stimulation cocktail containing phorbol 12-myristate 13-acetate (PMA) and ionomycin was used from the beginning of incubation to induce the strong, TCR-independent activation of effector T cells for intracellular cytokine detection. For anti-PD1 treatment, Pembrolizumab–KEYTRUDA^®^ (Merck Sharp & Dohme LLC, Rahway, NJ, USA) was added to a final concentration of 10 µg/mL, as described in other studies [25,26]. The cells were then incubated for 6 h at 37 °C in a 5% CO_2_ incubator. After incubation, the cells were characterized via flow cytometry, as previously described, using the anti-PD-1 panel described in Supplementary Table S1 and the gating strategy presented in Supplementary Figure S4.

### 2.5. Cytokine Quantification

Cytokine levels in the acellular fraction of MAF samples were quantified using the Human Macrophage/Microglia Panel Legendplex™ kit [BioLegend, cat no. 740502; IL-1β, IL-1RA, IL-4, IL-6, IL-10, IL-12p40, IL-12p70, IL-23, Arginase, CCL17(TARC), CXCL10 (IP-10), IFNγ, and TNFα] and the Human Anti-Virus Response Panel Legendplex™ kit [BioLegend, cat no. 740365; IL-1β, IL-6, IL-8, IL-10, IL-12; IFNα, IFNβ, IFNγ, IFNλ1, IFNλ2/3, TNFα, CXCL10 (IP-10), and GM-CSF], following the manufacturer’s instructions. Standards and experimental samples were acquired using an LSR II flow cytometer with FACS Diva software (version 6.1.3; BD Biosciences, USA). Data were analyzed using the LEGENDplex™ Data Analysis Software Suite (Biolegend, CA, USA).

### 2.6. Statistical Analysis

Statistical analyses were conducted using Statistical Package for the Social Sciences (SPSS) version 28 software (IBM, New York, NY, USA), and data visualization was performed with GraphPad Prism version 9 software (San Diego, CA, USA). Categorical variables were analyzed using the chi-square test, with Cramer’s V or Phi reported as measures of the effect size. Outliers were removed according to the ROUT method. Given the small sample size and non-normal distribution of variables, the Mann–Whitney U-test was used to compare worse vs. better prognosis groups, with *p*-values reported for each comparison and *r* reported as measures of the effect size. Multiple-comparison correction was performed using the Benjamini–Hochberg false discovery rate (FDR) procedure, applied separately within each figure/panel family of comparisons: (i) cytokines ( n = 20), (ii) innate immune parameters (n = 4), (iii) T cell subsets (n = 12), (iv) T cell granzyme subsets (n = 6), and (v) T cell exhaustion marker co-expression subsets (n = 14). The Wilcoxon signed-rank test was applied to assess differences between conditions within the same patient for each prognosis group. Binary logistic regression models were employed to evaluate associations, with odds ratios (ORs) and corresponding *p*-values reported for each variable. Differences were considered statistically different for *p*-values < 0.05. A summary of the statistical outputs and effect size is depicted in Supplementary Table S2.

## 3. Results

### 3.1. Cohort Characterization

Twenty-two women diagnosed with HGSC were enrolled in this study. MAF samples were collected at the time of diagnosis, prior to any chemotherapy treatment or surgery. Upon sample collection, patients undergo platinum therapy, with or without cytoreductive surgery, and the patient’s response to treatment and platinum-based therapy was recorded throughout their clinical course after chemotherapy (Supplementary Table S3). The cohort was stratified into two groups based on the TDR: patients with a TDR below six months were classified into the worse prognosis group, while those with a TDR of six months or more were classified into the better prognosis group. This six-month threshold was selected in accordance with established literature and clinical guidelines, where it is routinely applied to guide the management of disease recurrence following complete remission [27,28,29,30,31]. This cutoff was defined a priori, before any laboratory analysis was performed, based solely on the TDR; it was not derived from, or adjusted to, the immune profiling data. In OC, recurrence is defined as tumor reappearance after a complete response to first-line chemotherapy [32]. The interval between treatment completion and recurrence—commonly referred to as the platinum-free interval—is a clinically meaningful indicator of disease biology. Recurrence occurring within six months of treatment completion is associated with platinum-resistant disease, which is characterized by a poor response to subsequent therapy and limited survival outcomes [33]. It should be noted, however, that the TDR is a composite clinical outcome measure reflecting the overall disease trajectory—encompassing both the treatment response and untreated disease progression—rather than a direct surrogate for the platinum-free interval. Accordingly, the worse prognosis group should not be equated to a platinum-resistant group. The demographic and clinical characteristics of the patients are summarized in Table 1. The TDR differed significantly between the two prognosis groups (*p*-value < 0.001), as expected by design. All patients in the better prognosis group presented sensitivity to platinum therapy, in contrast to those in the worse prognosis group (*p*-value = 0.006); as both variables are closely linked to early disease progression, this association was expected and should not be interpreted as an independent validation of the prognostic grouping. Consistent with this, patients’ prognosis was dependent on their response to treatment.

### 3.2. Characterization of Cytokines and Inflammatory Mediators in MAF

**Figure 1:**
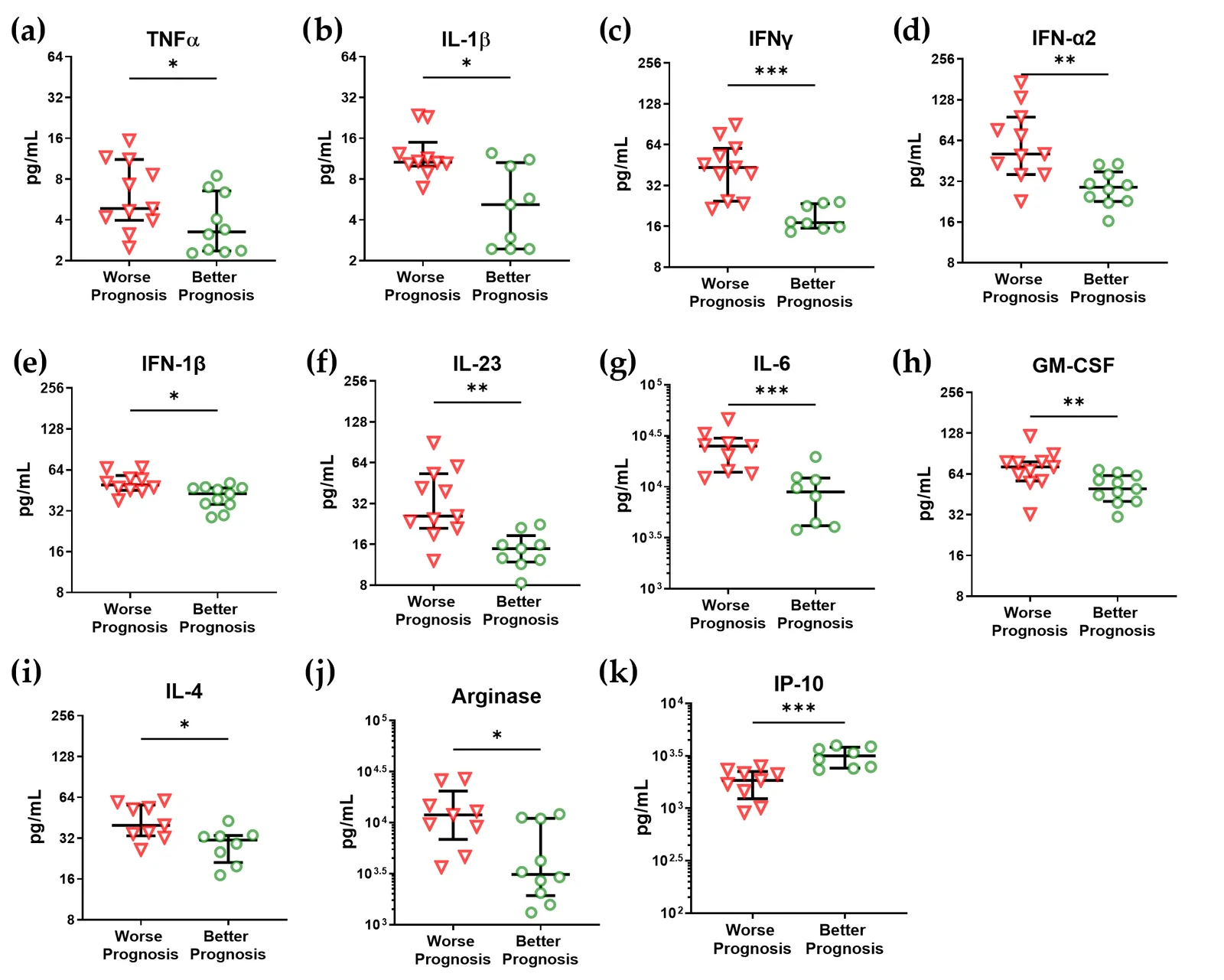
Characterization of immune mediators within the acellular fraction of MAF samples obtained from HGSC patients. For the analytes whose levels were statistically different between patients with worse (red) vs. better (green) prognosis, the absolute levels (pg/mL) were represented: TNFα (**a**), IL-1β (**b**), IFNγ (**c**), IFN-α2 (**d**), IFN-1β (**e**), IL-23 (**f**), IL-6 (**g**), GM-CSF (**h**), IL-4 (**i**), Arginase (**j**), and IP-10 (**k**). Each dot represents one biological replicate (individual patient sample). Data are shown in a scatter dot plot format as the median ± IQR. Statistical analysis was performed using a Mann–Whitney U-test. Statistically significant values are represented as follows: *, for *p* < 0.05; **, for *p* < 0.01; ***, for *p* < 0.001. Asterisks denote nominal Mann–Whitney U-test significance; FDR-adjusted *p*-values were calculated separately and are reported in Supplementary Table S2 and should be consulted for a multiplicity-corrected interpretation of these results.

The tumor microenvironment in OC is highly dynamic, with both pro-inflammatory and immunosuppressive cues contributing to disease progression. To better understand these interactions, the levels of key cytokines/chemokines and immune mediators were quantified in MAF samples from twenty-two treatment-naïve patients diagnosed with HGSC. Patients with a worse prognosis revealed statistically significant increases in the levels of TNFα (Figure 1a), IL-1β (Figure 1b), IFNγ (Figure 1c), IFN-α2 (Figure 1d), IFN-1β (Figure 1e), IL-23 (Figure 1f), IL-6 (Figure 1g), GM-CSF (Figure 1h), IL-4 (Figure 1i), and Arginase (Figure 1j) based on a nominal *p* < 0.05 from the Mann–Whitney U-test. Increased levels of IP-10 (Figure 1k) were also observed but in the patients with a better prognosis. Following correction for multiple comparisons using the Benjamini–Hochberg false discovery rate procedure, all associations described above remained statistically significant except that involving TNFα, which did not remain significant after correction (adjusted *p* = 0.058). This association should therefore be considered nominally significant and interpreted cautiously as an exploratory finding.

All other evaluated analyte levels were not statistically significant (IL-1RA, IFN-λ1, IFN-λ2/3, IL-8, IL-10, IL-12p40, IL-12p70, and TARC). The cytokine profiling of treatment-naïve MAF supernatants suggests that a combined pro-inflammatory and immunosuppressive environment correlates with an unfavorable prognosis in patients with HGSC.

### 3.3. Immunophenotyping of Innate Cells from MAF Samples of HGSC Patients

**Figure 2:**
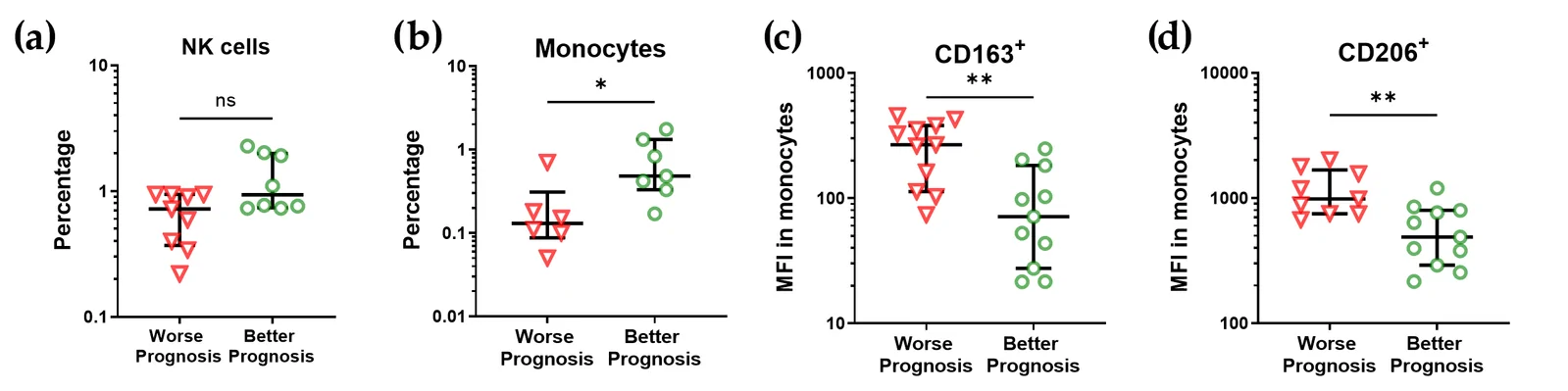
Immunophenotyping of innate immune cells present in MAF samples obtained from HGSC patients. Percentage of NK cells ((**a**); CD3-CD56+) and monocytes ((**b**); CD66b-CD3-CD56-CD19-HLA-DR+) present in MAF samples of the patients with worse (red) and better (green) prognosis. The Mean Fluorescence Intensity (MFI) of CD163 (**c**) and CD206 (**d**) was evaluated within the monocyte population. The gating strategy and representative plots are depicted in Supplementary Figure S1. Each dot represents one biological replicate (individual patient sample). Data are shown in a scatter dot plot format as the median ± IQR. Statistical analysis was performed using a Mann–Whitney U test. Statistically significant values are represented as follows: *, for *p*  <  0.05; **, for *p*  <  0.01; ns—non statistical difference.

Innate immune cells, including monocytes, macrophages, and natural killer (NK) cells, are key components of the MAF environment and are often reprogrammed to support tumor growth [34]. Building on the previous analysis of cytokine and inflammatory mediator profiles, the innate cell populations of MAF were characterized. A tendency for a decrease in the percentages of NK cells was observed in patients with worse prognosis (Figure 2a). The worse-prognosis group presents fewer monocytes (Figure 2b) overall but with a more pronounced M2-like (pro-tumoral) polarization phenotype (higher CD163/CD206 intensity per cell), represented in Figure 2c,d, rather than a higher absolute number of polarized cells. These findings further highlight the immunosuppressive environment associated with poor clinical outcomes.

### 3.4. Evaluation of T Cell Phenotypes in MAF Samples of HGSC Patients

**Figure 3:**
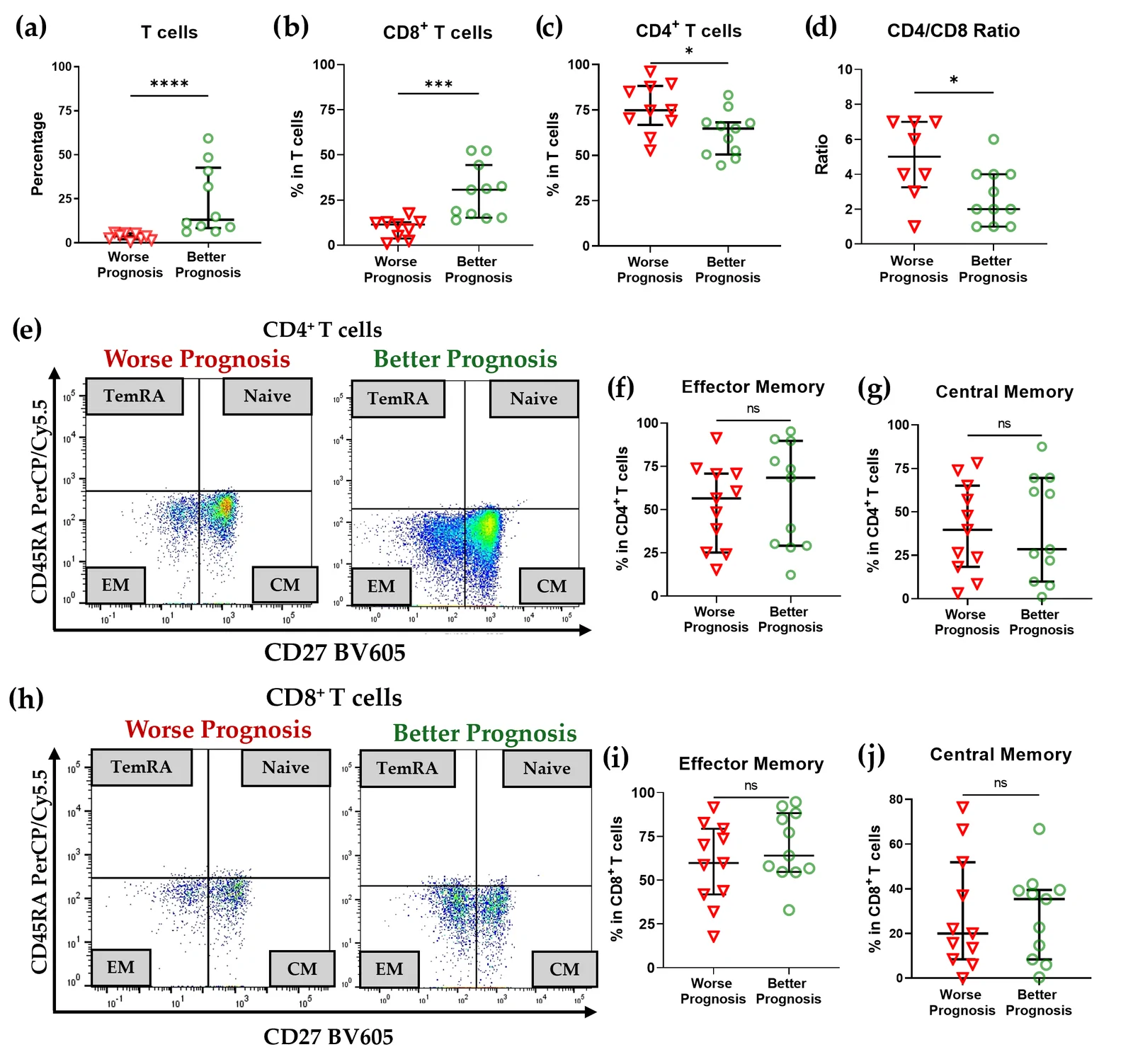
Characterization of T cells from MAF samples obtained from HGSC patients. The percentages of T cells, (**a**), CD8^+^ (**b**), CD4^+^ (**c**), and the CD4/CD8 ratio (**d**), are reported. The dot plots are representative of the gate of CD27 with CD45RA from patients HGSC#10 (worse prognosis) and HGSC#11 (better prognosis) for CD4^+^ (**e**), along with the percentage of effector memory CD4^+^ (**f**) and central memory CD4^+^ (**g**) T cells. Also, the same representative dot plots are used for CD8^+^ (**h**), along with the percentage of effector memory CD8^+^ (**i**) and central memory CD8^+^ (**j**) T cells present in the MAF samples. Samples of the patients with worse (red) and better (green) prognosis are represented in a scatter plot. Each dot represents one biological replicate (individual patient sample). Data are shown as the median ± IQR. Statistical analysis was performed using a Mann–Whitney U test. Statistically significant values are represented as follows: *, for *p*  <  0.05; ***, for *p*  <  0.001; and ****, for *p*  <  0.0001; ns—non statistical difference. Asterisks denote nominal Mann–Whitney U-test significance; FDR-adjusted *p*-values were calculated separately and are reported in Supplementary Table S2 and should be consulted for a multiplicity-corrected interpretation of these results. Naïve (CD45RA^−^CD27^+^), central memory (CM; CD45RA^−^CD27^+^), effector memory (EM; CD45RA^−^CD27^−^), and terminally differentiated CD45RA-expressing memory T cells (TemRA; CD45RA^+^CD27^−^).

T cells are central to the immune response against tumors, yet their function can be severely compromised within the tumor microenvironment [20]. To further investigate the role of T cells in HGSC, we characterized the T cell populations present in the MAF of these patients. Patients with a worse prognosis had a significantly decreased percentage of T cells (Figure 3a), specifically CD8^+^ T cells (Figure 3b). Additionally, patients with a worse prognosis presented a higher percentage of CD4^+^ T cells (Figure 3c), which consequently led to a higher CD4/CD8 ratio (Figure 3d). T cells were further evaluated for the expression of CD27 and CD45RA to assess naïve and memory subsets (Figure 3e,h). However, no statistically significant differences were observed in the percentages of effector (CD45RA^−^CD27^−^) and central memory (CD45RA^−^CD27^+^) cells between the worse and better prognosis patient groups, in either CD4^+^ (Figure 3f,g) or CD8^+^ T cells (Figure 3i,j). Following a correction for multiple comparisons, the differences in CD8^+^ T cells, CD4^+^ α4β7^+^, CD8^+^ Eomes^+^, and CD8^+^ α4β7^+^ T cells remained statistically significant, whereas the CD4/CD8 ratio and CD4^+^ Eomes^+^ T cell findings did not survive the correction (adjusted *p* = 0.069 and *p* = 0.065, respectively) and should therefore be interpreted as nominally significant, exploratory findings. No naïve (CD45RA^−^CD27^+^) or terminally differentiated (CD45RA^+^CD27^−^) CD4^+^ or CD8^+^ T cells were detected in MAF samples from HGSC patients.

**Figure 4:**
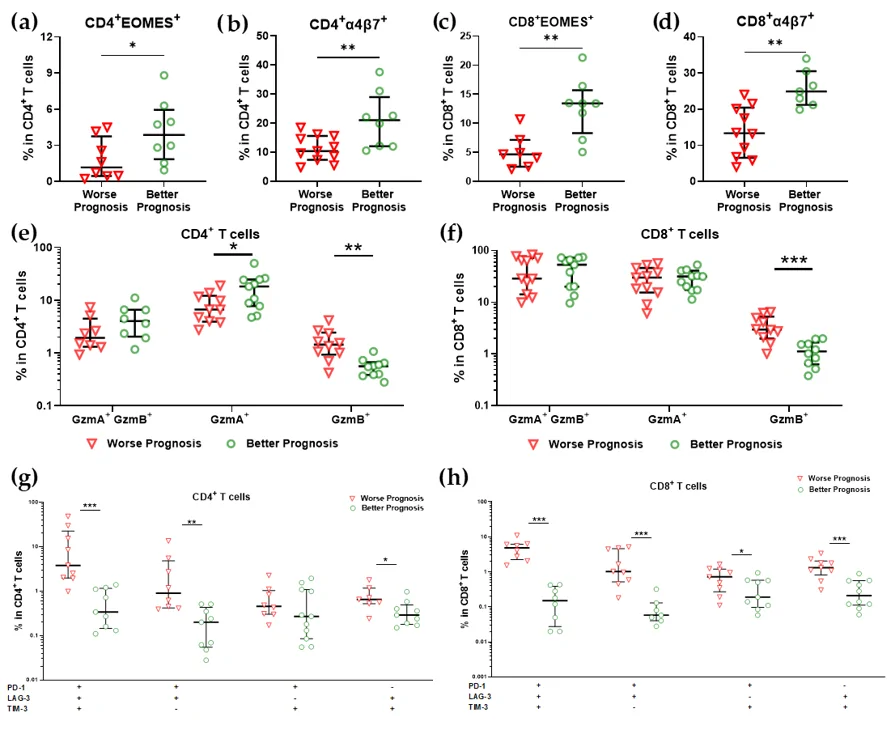
Evaluation of activation and exhaustion profiles of T cells from MAF obtained from HGSC patients. Percentages of the activation marker Eomesodermin (EOMES) and of the homing integrin α4β7 in CD4^+^ ((**a**) and (**b**), respectively) and CD8^+^ T cells ((**c**) and (**d**), respectively). Percentage of granzymes A and/or B in CD4^+^ (**e**) and CD8^+^ (**f**) T cells. Percentage of the exhaustion markers PD-1, LAG-3, and TIM-3 present in CD4^+^ (**g**) and CD8^+^ T cells (**h**). Each dot represents one biological replicate (individual patient sample). Gating strategies and representative plots are depicted in Supplementary Figures S3 and S4. Samples of the patients with worse (red) and better (green) prognosis are represented in a scatter plot format as the median ± IQR. Statistical analysis was performed using a Mann–Whitney U-test. Statistically significant values are represented as follows: *, for *p* < 0.05; **, for *p* < 0.01; ***, for *p* < 0.001. Asterisks denote nominal Mann–Whitney U-test significance; FDR-adjusted *p*-values were calculated separately and are reported in Supplementary Table S2 and should be consulted for a multiplicity-corrected interpretation of these results.

We further analyzed the expression of T cell markers associated with activation (Eomesodermin, EOMES), homing (α4β7 integrin), and exhaustion receptors (PD-1, LAG-3, and TIM-3), as well as effector molecules (granzyme A and B). Generally, HGSC patients with a worse prognosis exhibit a CD4^+^ and CD8^+^ T cell-exhaustion-associated phenotype, as opposed to the activated T cell profile observed in patients with a better prognosis. Specifically, patients with a better prognosis present higher percentages of T cells with an activated profile and expressing homing receptors, both in CD4^+^ (Figure 4a,b) and CD8^+^ T cells (Figure 4c,d). Additionally, the percentage of CD4^+^ T cells expressing granzyme A (GzmA) (Figure 4e) is higher for patients with a better prognosis, while the opposite occurs for the expression of GzmB (Figure 4e). Similarly, the percentage of CD8^+^ T cells expressing GzmB is lower in patients with a better prognosis (Figure 4f). Regarding the T cell-exhaustion-associated phenotype, the worse prognosis group consistently exhibited higher percentages of CD4^+^ (Figure 4g) and CD8^+^ (Figure 4h) T cells expressing exhaustion markers, specifically the following populations: PD-1^+^LAG-3^+^TIM-3^+^, PD-1^+^LAG-3^+^TIM-3^−^, and PD-1^−^LAG-3^+^TIM-3^+^ in both CD4^+^ and CD8^+^ T cells; and PD-1^+^LAG-3^−^TIM-3^+^, PD-1^+^LAG-3^−^TIM-3^−^, and PD-1^−^LAG-3^−^TIM-3^+^ were found exclusively in CD8^+^ T cells (Supplementary Figure S5). Overall, these results highlight an exhausted profile of T cells in the worse prognosis group compared with the better prognosis group, suggesting this characterization as a potential biomarker candidate, at the diagnostic stage.

### 3.5. Binary Logistic Regression Model to Predict Disease Progression at Diagnosis

Our findings suggest that the extent of the T cell-exhaustion-associated phenotype may serve as a useful prognostic indicator, as patients with a higher percentage of exhausted T cells at diagnosis are more likely to have worse clinical outcomes. To evaluate this, we developed a predictive model for patient prognosis based on MAF analysis, including the percentage of CD8^+^ and CD4^+^ T cells expressing the three inhibitory markers (PD-1^+^TIM-3^+^LAG-3^+^) and the levels of IL-23, a key cytokine involved in maintaining chronic inflammation (Table 2). These variables were selected based on their high effect sizes when comparing the worse and better prognosis groups (Supplementary Table S2). This predictive model followed a binary logistic regression, based on the Nagelkerke R^2^ correlation. The model was significant (*p* < 0.001) with a Nagelkerke R^2^ of 0.73. In this model, the outcome variable was coded as prognosis = 1 (good prognosis), representing the event of interest, whereas prognosis = 0 (worse prognosis) was used as the reference category. While the individual variables did not contribute significantly on their own, combining the three variables enabled the preliminary association of patient outcomes at diagnosis.

Using the predictive model, a Receiver Operating Characteristic (ROC) curve was developed with the probabilities calculated via binary logistic regression, revealing an area under the curve–AUC = 0.955, with a 95% CI of 0.86–1.00 (obtained using the Hanley–McNeil method), and a *p*-value ≈ 0.0005 (Supplementary Figure S6). This model presents high sensitivity (91%) and specificity (80%). Using the standard probability threshold of 0.5, it correctly identifies 10 of the 11 patients with a better prognosis (sensitivity = 91%) and correctly classifies 8 of the 10 patients with a worse prognosis (specificity = 80%). However, to obtain an unbiased estimate of model performance, we performed leave-one-out cross-validation (LOOCV). The cross-validated AUC was 0.764, substantially lower than the apparent AUC of 0.955, suggesting potential overfitting due to the small sample size. Therefore, these findings should be interpreted with caution, and external validation based on larger, independent cohorts is essential to confirm the model’s robustness and generalizability.

### 3.6. Ex Vivo PD-1 Blockade Enhances Effects or Functions in MAF-Derived T Cells from Patients with HGSC

**Figure 5:**
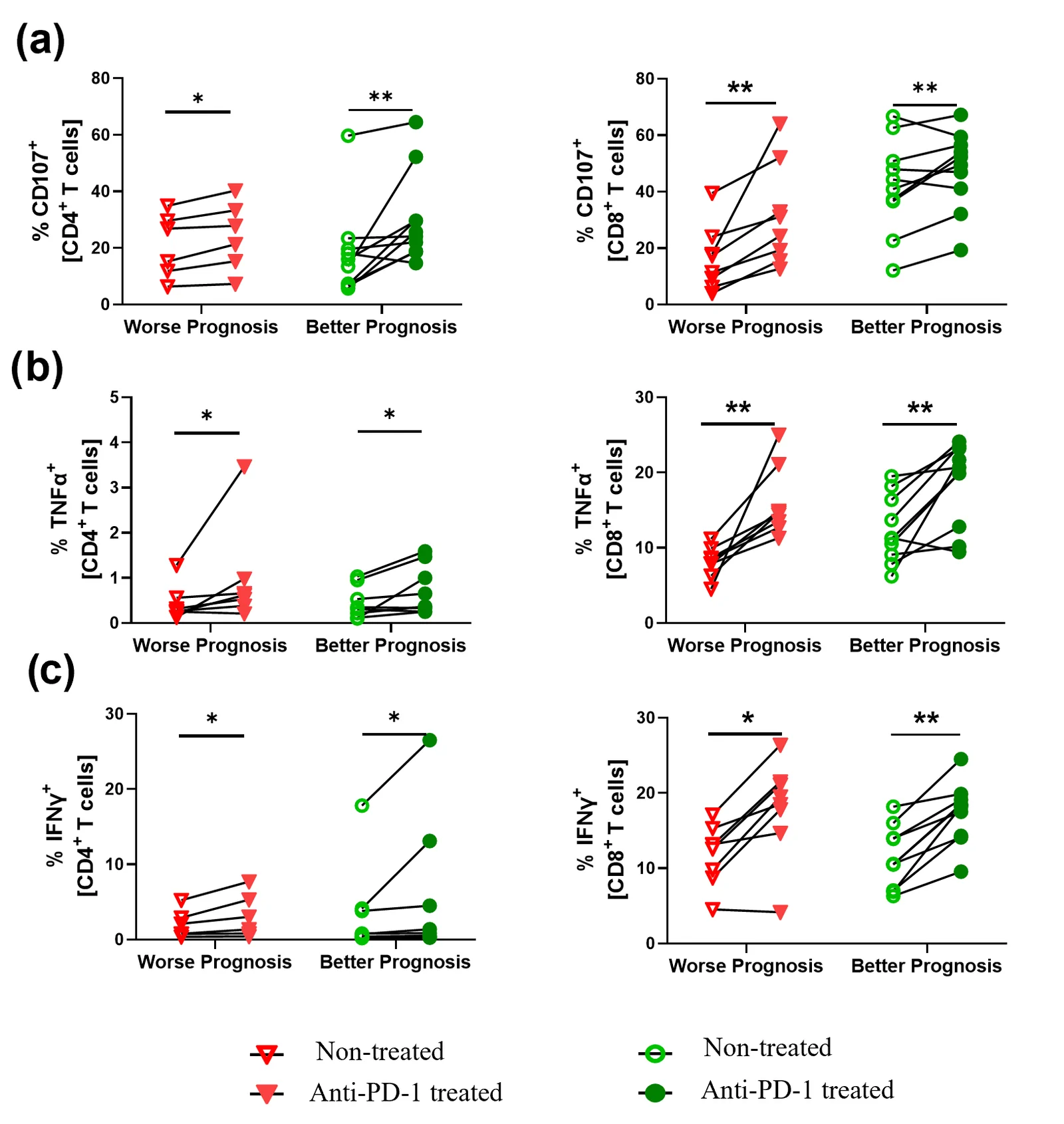
Intracellular production of IFNγ and TNFα and CD107 expression in MAF-derived T cells following ex vivo stimulation and treatment with anti-PD-1 (Pembrolizumab). MAF-derived cells were cultured under activating conditions (PMA/ionomycin) and treated with anti-PD-1 or left untreated. Expression of CD107 in CD4^+^ and CD8^+^ T cells (**a**), and intracellular levels of TNFα (**b**) and IFNγ (**c**) in CD4^+^ and CD8^+^ T cells in anti-PD-1-treated MAF-derived cells compared with the non-treated cells. Gating strategies and representative plots are depicted in Supplementary Figure S4. Solid black lines connect the same patient under the two conditions. Statistical analysis was performed using the Wilcoxon signed rank test. Statistically significant values are represented as follows: *, for *p*  <  0.05; and **, for *p*  <  0.01.

To evaluate the functional competence of T cells present in MAF from patients with HGSC, ex vivo stimulation assays were performed using PD-1 immune checkpoint blockade. MAF-derived cells were cultured in the presence or absence of the anti-PD-1 monoclonal antibody Pembrolizumab for 6 h. To determine whether PD-1 blockade could restore effector functions in exhausted-like T cells, the degranulation capacity of T cells (CD107 expression) and the intracellular cytokine production were assessed following treatment. Pembrolizumab exposure resulted in a significant increase in the percentage of CD107^+^ cells (Figure 5a) and in the production of the pro-inflammatory cytokines TNFα (Figure 5b) and IFNγ (Figure 5c) compared with untreated samples. This enhancement was observed in both CD4^+^ and CD8^+^ T cell subsets and was consistent across both groups.

## 4. Discussion

This study explores the dynamics of the TIME in the MAF of HGSC patients. Patients were stratified according to prognosis, revealing that those with a worse prognosis exhibited a more pro-inflammatory and immunosuppressive TIME compared to the patients with a better prognosis. Notably, the worse prognosis group presented reduced percentages of T cells (specifically CD8^+^) and higher expression of exhaustion markers (PD-1, TIM-3, and LAG-3). These findings suggest a strong association between the T cell-exhaustion-associated phenotype and unfavorable clinical outcomes. To counteract this exhausted state, MAF-derived T cells were treated with Pembrolizumab. The treatment significantly enhanced the production of TNFα and IFNγ by CD4^+^ and CD8^+^ T cells, suggesting an ex vivo functional response to PD-1 blockade. Importantly, these functional improvements were observed across all patient groups, regardless of prognosis, as elevated PD-1 expression was consistently detected among them. These results suggest that this immunotherapeutic strategy may improve T cell efficacy and potentially reverse the exhaustion-associated phenotype in the context of HGSC. The cytokine and chemokine analysis in the acellular fraction further highlighted the immunosuppressive and inhospitable nature of the MAF microenvironment. Changes in cytokines can induce subtle yet impactful modifications to the TIME, ultimately interfering with the immune system’s ability to coordinate an effective anti-tumor response [35]. This immunosuppressive microenvironment likely contributes to the observed decrease in the T cell percentage within the MAF [36]. Several studies have established that lower percentages of T cells at diagnosis are significant predictors of poor survival outcomes in various cancers, including lymphoma, bladder, muscle-invasive, and colorectal cancer [37,38,39]. In OC, reduced T cell levels can serve as a valuable predictive biomarker, aiding the identification of patients who might benefit from treatments aimed at restoring antitumor immunity [35]. Our study corroborates these findings, as patients with a worse prognosis presented with lower percentages of T cells. Also, even though single GzmB^+^ T cells represented a minor population in our samples, they may nonetheless contribute substantially to immune activity within the tumor microenvironment [40,41,42]. The divergent expression of GzmA and GzmB observed in CD4^+^ T cells—a higher percentage of GzmA^+^ and lower percentage of GzmB^+^ T cells in patients with a better prognosis—likely reflects distinct activation and differentiation states rather than absence of biological relevance. Elevated percentages of GzmB^+^ cells in patients with a poor prognosis may indicate the accumulation of terminally differentiated or chronically stimulated T cells that retain GzmB but display functional exhaustion [43]. Together, these findings suggest that differential granzyme profiles mirror distinct T cell states associated with clinical outcomes in HGSC.

The immunosuppressive TIME in OC supports T cell exhaustion and weakens the effector function of T cells [44]. It has been demonstrated that cancer cells directly induce T cell exhaustion through cell-to-cell crosstalk [45]. Within the TIME, exhausted T cells exhibit upregulated suppressive receptors that prominently suppress antitumor immune responses while reinforcing the immunosuppressive TIME [21]. The elevated numbers of exhausted T cells in OC patients are positively correlated with poor prognostic outcomes, suggesting that restoring the T cell effector function by targeting suppressive receptors could be a potential therapeutic strategy [46].

Our model shows the association between exhausted T cells and poorer patient prognosis, as well as increased IL-23 levels, in the MAF of patients with a worse prognosis. IL-23 is a crucial cytokine that promotes tumorigenesis, with elevated levels linked to a worse prognosis in many human carcinomas [47,48]. In a colorectal cancer model, mice deficient in IL-23 or its receptor exhibited reduced tumor multiplicity and growth [49], suggesting the potential application of anti-IL-23 therapies in cancer treatment. A possible therapeutic strategy involves combining anti-CD40L and anti-IL-23 monoclonal antibodies to enhance antitumor efficacy in various carcinomas [50]. This implies that, in the future, anti-IL-23 could potentially be repurposed for use in immuno-oncology alongside other immunotherapies. Although none of the individual variables included in our predictive model were independently significant, their combination resulted in a significant and exploratory predictive model. One of the main limitations of this study is the small sample size, reflecting the lower incidence of OC. This limitation likely contributes to the absence of significance of individual variables and could potentially be addressed with an increased sample size in future studies.

Ovarian tumors are infiltrated by PD-1^+^ TIM-3^+^ T cells with partial-exhaustion features, suggesting that the TIME plays a role in maintaining this specific phenotype [51]. Tumor cells express tumor-associated antigens capable of stimulating TAMs, providing both co-stimulatory and co-inhibitory signaling for TAMs and T cells, respectively. MAF from OC patients comprises the abundant chemokine C-C Motif Chemokine Ligand 23 (CCL23). TAMs are critical players within the TIME, facilitating tumor development and metastasis. CCL23, secreted by macrophages, drives the metastatic potential of OC cells through C-C chemokine receptor type 1 (CCR1) and produces an exhausted T cell phenotype by upregulating inhibitory markers on the CD8^+^ T cell population, including LAG-3 and TIM-3 [52]. Our results suggest that patients with a worse prognosis exhibit an increased pro-tumoral macrophage phenotype and significantly higher expression of LAG-3 and TIM-3 markers on T cells compared to patients with a better prognosis.

Cancer cells often metastasize through epithelial–mesenchymal transition (EMT) [53]. Tumors with infiltrated or inflamed microenvironments tend to have more mesenchymal cells than excluded or desert tumors [54]. While the high presence of CD8^+^ T cells is associated with longer survival rates, mesenchymal tumor cells tend to be more resistant to anti-tumor immune responses [44]. The expression of Galectin-3 (*LGALS3* gene) is strongly linked to EMT. In tumors with mesenchymal OC cells, there is significant interaction through the LGALS3–LAG3 axis in CD8^+^ T cells, which exhibit elevated LAG-3 levels, a well-established marker of T cell exhaustion [54]. Mesenchymal OC cells may facilitate CD8^+^ T cell exhaustion via the LGALS3–LAG3 axis [54]. In our cohort, patients with a worse prognosis presented higher expression of LAG-3 in CD8^+^ T cells compared to patients with a better prognosis. This finding suggests that the exhaustion of these cells can be driven, at least in part, by the LGALS3–LAG3 axis. Nevertheless, further functional assays would be necessary to confirm this theory.

The PD-1/PD-L1 axis results in the inactivation and depletion of T cells, mediating tumor-induced immune escape, which plays a crucial role in the TIME of OC. This axis is an ideal target for reversing the immunosuppressive TIME [55]. However, in OC, PD-L1 expression is notably lower compared to other immunosensitive tumors, which helps explain the limited efficacy of anti-PD-1/PD-L1 treatments in this type of cancer [56]. Recently, a clinical trial with advanced OC showed promising results in the use of an anti-PD-1 antibody (Pembrolizumab) for frontline therapy [57]. In our study, the expression of three inhibitory molecules—PD-1, LAG-3, and TIM-3—was increased in both CD4^+^ and CD8^+^ T cells in patients with a poor prognosis, suggesting a direct relationship between worse clinical outcomes and a T cell-exhaustion-associated phenotype. An assessment of cytokine production (IFNγ and TNFα) remains the classical approach for evaluating T cell function. However, to precisely elucidate the mechanisms underlying the reverted phenotype, additional assays are required—such as cytotoxicity assays, T cell proliferation measurements, and the longitudinal tracking of exhaustion reversal.

In OC, tumor-infiltrating CD8^+^ T cells accumulate in tumor islets, engaging antigens and upregulating PD-1 expression, which restrains their effector function [58]. Despite this, PD-1^+^ CD8^+^ T cells within the epithelium may retain multifunctional capacity, although they exhibit a sustained state of exhaustion. This exhaustion is influenced by varying levels of CD28 co-stimulation, provided by antigen-presenting cells (APCs) within the intraepithelial tumor myeloid niche [58]. CD28 co-stimulation plays a critical role in enhancing the effector function of exhausted CD8^+^ T cells, enabling their activation when PD-1 is blocked [58]. However, this response depends on the presence of tumor myeloid APCs. Exhausted T cells lacking proper CD28 co-stimulation fail to respond to anti-PD-1 therapy [58,59,60]. Therefore, combining PD-1 blockade with approaches that enhance CD28 signaling or improve APC function may offer a promising therapeutic strategy to restore T cell functionality and improve outcomes in OC patients.

Our study, consistent with others [21], identified the abundant expression of co-inhibitory receptors on T cells derived from the MAF of HGSC patients. This underscores the potential of checkpoint blockade as a promising strategy for enhancing functional tumor surveillance in this cancer type. After confirming the pronounced exhaustion-associated phenotype among T cells, we evaluated the impact of PD-1 blockade on their functionality, indicated by increased production and release of the cytokines IFNγ and TNFα. Despite variations among the analyzed MAF samples, including differences in TIMEs, phenotypic characteristics, and the overall cellular composition (such as the presence of tumor cells and APCs), however, additional variability was introduced by the cryopreservation process, since freeze–thaw cycles can influence T cell function, marker expression, and cytokine responsiveness; nonetheless, post-thaw viability across samples averaged approximately 90%, supporting the reliability of the functional readouts obtained.

The enhancement of T cell effector function following ex vivo PD-1 blockade suggests that the MAF microenvironment can be therapeutically reprogrammed. Clinically, this could be achieved via the intraperitoneal administration of anti-PD-1 antibodies, which provides high local antibody concentrations within the peritoneal cavity and has shown feasibility in early clinical studies of peritoneal malignancies [61]. Since systemic CD28 agonism is associated with cytokine-release toxicity, alternative co-stimulatory strategies such as tumor-targeted [62] or CD27 agonists [63] or APC-activating agents (e.g., CD40L agonists) may offer safer means of reproducing the co-stimulatory effects observed ex-vivo [64]. Moreover, regional adoptive T cell approaches, including the intraperitoneal infusion of activated or engineered T cells in combination with local PD-1 blockade, could further enhance T cell reinvigoration in the MAF niche [65]. Future translational work should focus on IP dose finding, cytokine monitoring, and biomarker-based patient selection guided by the exhaustion and cytokine signatures identified in this study.

This study opens several avenues for future research to validate and extend our findings. Nevertheless, the relatively small sample size precluded the development of a fully adjusted multivariable model incorporating all clinically relevant covariates, limiting the ability to account for potential confounding factors. Future studies with larger cohorts are therefore warranted to strengthen the robustness and generalizability of these observations. Further mechanistic investigations should include functional assays, including cytotoxicity testing, T cell proliferation analysis, and the longitudinal monitoring of exhaustion reversal, to provide direct evidence of T cell functional restoration. Regarding the ex vivo Pembrolizumab assay, although a parallel isotype control was not included in the present design, comparable ex vivo/in vitro Pembrolizumab functional assays have consistently reported that isotype control antibodies induce no measurable functional effect relative to untreated conditions, supporting the interpretation of our findings [66,67]; nonetheless, future studies should incorporate a parallel isotype control to further strengthen the attribution of the observed effects specifically to PD-1 blockade. An assessment of PD-L1 expression in tumor cells from MAF samples using multiplex immunohistochemistry would allow the simultaneous evaluation of CD4, CD8, PD-1, and PD-L1 expression within the same tissue context. Additionally, the use of in vivo models represents a relevant next step to validate the ex vivo observations and to evaluate the translational potential of the identified immune pathways.

## 5. Conclusions

This study highlights three key findings that warrant further investigation. First, the TIME in MAF potentiates tumor growth by contributing to an immunosuppressive environment. This environment often correlates with patient prognosis, influencing both the effectiveness of the immune response and the potential for disease progression. Secondly, patients with a worse prognosis exhibit lower percentages of T cells at diagnosis, and these cells have an exhaustion-associated phenotype. Finally, ex vivo PD1 blockade enhanced cytokine production by patient-derived T cells displaying an exhaustion-associated phenotype. These findings highlight how PD-1 inhibitors can revitalize exhausted-like T cells. Furthermore, they suggest a pathway for investigating additional immune checkpoint inhibitors, including anti-TIM-3 and anti-LAG-3 monoclonal antibodies, as complementary treatments. These agents could further reverse T cell exhaustion and strengthen the antitumor immune response in the TIME of OC patients. Combining these strategies might create new therapeutic opportunities to enhance outcomes for patients with HGSC, although validation based on a larger, independent cohort and, ideally, correlations with clinical responses to checkpoint inhibition are crucial.

## Figures and Tables

**Table 1:** Demographic and clinical characteristics of malignant ascitic fluid (MAF) samples obtained from high-grade serous carcinoma (HGSC) patients.

	Worse Prognosis (n = 11)	Better Prognosis (n = 11)	*p*-Value ^1^	Effect Size ^2^
Demographic characteristics				
Age (years), median (IQR)	74 (43)	70 (31)	0.534	0.200
Clinical characteristics				
TDR (months), median (IQR)	3 (4)	13 (27)	<0.001	0.554
Patient status			0.135	0.426
DOD, % (n)	54.5 (6)	27.3 (3)		
AWD, % (n)	45.5 (5)	45.5 (5)		
AWoD, % (n)	0 (0)	27.3 (3)		
Cytoreductive surgery during follow-up			0.170	0.293
Yes, % (n)	18.2 (2)	45.5 (5)		
No, % (n)	81.8 (9)	54.5 (6)		
Platinum response			0.006	0.683
Sensitive, % (n)	36.4 (4)	100 (11)		
Refractory, % (n)	18.2 (2)	0 (0)		
NA, % (n)	45.5 (5)	0 (0)		
Treatment response			0.027	0.705
No treatment, % (n)	27.3 (3)	0 (0)		
Negative, % (n)	18.2 (2)	0 (0)		
Partial, % (n)	36.4 (4)	81.8 (9)		
Complete, % (n)	0 (0)	18.2 (2)		
NA, % (n)	18.2 (2)	0 (0)		

^1^ Qui-Square test; ^2^ Cramer’s V or Phi; patient status: AWD—alive with disease; AWoD—alive without disease; DOD—died of disease. IQR—interquartile range; NA—not available; TDR—time to death or recurrence.

**Table 2:** Binary logistic regression, based on the Nagelkerke R2 correlation, to predict patients’ prognosis.

Predictor Variables	Binary Logistic Regression Model (n = 21) (*p*-Value ≤ 0.001; R^2^ = 0.728)		
	Adjusted OR	*p*-Value	95% CI for Exp (B)
CD8^+^ PD-1^+^ TIM-3^+^ LAG-3^+^ T cells (%)	0.841	0.824	0.182–3.873
CD4^+^ PD-1^+^ TIM-3^+^ LAG-3^+^ T cells (%)	0.616	0.467	0.167–2.276
IL-23 (pg/mL)	0.910	0.267	0.771–1.075
Constant	42.504	0.033	-

## Data Availability

The data presented in this study are available in the main text and Supplementary Material.

## Supplementary Materials

The following supporting information can be downloaded at: https://www.mdpi.com/article/10.3390/cancers18162612/s1. Table S1. Antibody panels used to characterize the cellular fraction of malignant ascitic fluid (MAF) samples. Table S2. Statistical outputs and sample size calculation. Table S3. Demographic and clinical characteristics of malignant ascitic fluid (MAF) samples collected from patients with high-grade serous carcinoma (HGSC). Figure S1. Gating strategy for flow cytometry analysis of innate populations. The first gating step was to exclude most dead cells, debris, and platelets. Singlets were selected for posterior analysis, and neutrophils (CD66b+) were excluded. For the analysis of myeloid cells, natural killer (NK, CD56+), NKT (CD3+ CD56+), and T (CD3+) cells were excluded, followed by the exclusion of B cells (CD19+). Monocytes were gated on HLA-DR-positive cells. The surface expression of CD86, HLA-DR, CD163, and CD206 was quantified in the total monocyte population. This gating strategy illustrates patient HGSC#5. Figure S2. Gating strategy for flow cytometry analysis of adaptative populations. The first gating step was to exclude most dead cells, debris, and platelets. After that, singlets were selected for subsequent analysis. B cells were identified by CD20 expression, and T cells by CD3. T cells were further gated on CD8 and CD4, to identify the CD8+ and CD4+ T cells, respectively. The CD4+ and CD8+ T cells were also gated on CD45RA and CD27 to quantify the naïve, effector, central, and terminally differentiated CD45RA-expressing memory cells (TemRA). T cell activation was assessed by Eomes and homing of T cells by integrin α4β7 expression (representative plots in CD8+ T cells). This gating was performed using the sample from patient HGS#11. Figure S3. Gating strategy for flow cytometry analysis of T cell exhaustion. The first gating step was to exclude most dead cells, debris, and platelets. Afterward, singlets were selected for posterior analysis. CD4+ T cells were identified with CD4 and CD3 markers, and CD8+ T cells with CD8 and CD3 markers. The exhaustion state of T cells was then measured using the markers PD-1, LAG-3, and TIM-3. Additionally, we measured the intracellular expression of granzymes A (Gran. A) and B (Gran. B) on CD4+ and CD8+ T cells (representative plots for CD8+ T cells). This gating was performed with the sample from patient HGS#27. Figure S4. Gating strategy for flow cytometry analysis of T cell stimulation. Upon being stimulated in vitro, cells were stained using the “stimulation of T cells” panel. For the analysis, the first gating step was to exclude most dead cells, debris, and platelets. Afterward, singlets were selected for posterior analysis. CD4+ T cells were assessed with CD4 marker, and CD8+ T cells with a CD8 marker, within the CD3 positive cells. Then, we evaluated the intracellular expression of cytokines IFNγ and TNFα by the CD4+ and CD8+ T cells (representative plots for unstimulated CD8+ T cells). This gating was performed with the sample from patient HGS#39. Figure S5. Percentage of the exhaustion markers PD-1, LAG-3 and TIM-3 present in CD4+ (**a**) and CD8+ T cells (**b**). Each dot represents one biological replicate (individual patient sample). Gating strategies and representative plots depicted in Supplementary Figures S3 and S4. Samples of the patients with worse (red) and better (green) prognosis are represented in a scatter plot format as median ± IQR. Statistical analysis was performed using a Mann–Whitney U test. Statistically significant values are represented as: *, for *p* < 0.05; **, for *p* < 0.01; and ***, for *p* < 0.001. Figure S6. Receiver Operating Characteristic (ROC) curve for the regression model using the probabilities resulting from the binary logistic regression model. The area under the curve (AUC) value for our predictive model is 0.955 (95% CI of 0.86–1.00) with a *p*-value ≈ 0.0005. The sensitivity and specificity are 91% and 80%, respectively. The LOOCV is 0.764. In the logistic regression model, the outcome variable was coded as Prognosis = 1 (Good Prognosis), representing the event of interest, whereas Prognosis = 0 (Worse Prognosis) was used as the reference category.

## Abbreviations

The following abbreviations are used in this manuscript:

HGSC	High-grade serous carcinoma
IL	Interleukin
IFNγ	Interferon gamma
LAG-3	Lymphocyte activation gene 3 protein
MDSCs	Myeloid-derived suppressor cells
MAF	Malignant ascitic fluid
OC	Ovarian cancer
PD-1	Programmed cell death protein 1
TAMs	Tumor-associated macrophages
TCR	T cell receptor
TDR	Time to death or recurrence
TILs	Tumor-infiltrated lymphocytes
TIM-3	T cell immunoglobulin and mucin domain-containing protein 3
TIME	Tumor immune microenvironment
TNFα	Tumor necrosis factor-α
Tregs	Regulatory T cells
